# The present status of radiopharmaceuticals in the country

**Published:** 2016

**Authors:** Amir Reza Jalilian, Davood Beiki


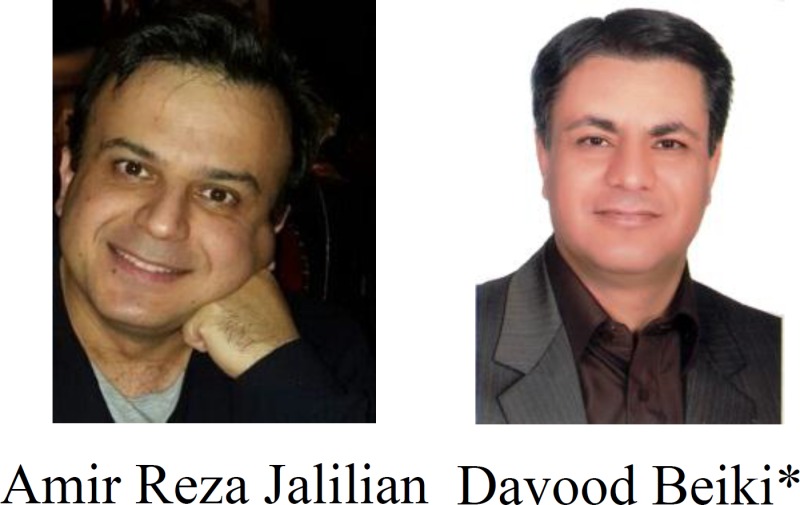


Radiopharmaceuticals are the major and essential component in nuclear medicine and are the most prominent aspect of peaceful applications of nuclear sciences in human being’s daily life. These radioactive materials are formulated in appropriate forms for administration to human being for the diagnosis and the therapy of human diseases. Diagnostic radiopharmaceuticals are the majorly applied radiopharmaceuticals worldwide (>90%), while therapeutic applications are still in the growing process.

Production of radiopharmaceuticals almost started in 1980’s in Iran with the fabrication of ^99^Mo/^99m^Tc generators, the most widely used component in the nuclear medicine practice. Since then, many Tc-99m kits for single photon emission computed tomography (SPECT) were prepared and developed in the country together with other diagnostic and therapeutic radiopharmaceuticals in last 3 decades. Recently, positron emission tomography (PET) has been initiated in the country using the most important PET tracer, i.e. ^18^F-FDG. The advances of radiopharmaceutical sciences in Iran have been recently reported at international levels (1), with great position compared to other countries in the Middle East (2). In this editorial, the status of production and availability of radiopharmaceuticals in the country has been presented. Although the research and development of radiopharmaceuticals has a long history in the country, however, the initiation of *National Iranian Radiopharmaceutical Project *in 2010 led to initiation of many new pre-clinical/clinical evaluations pushing the developed radiopharmaceuticals into clinical applications and market (3).

Tc-99m is used in more than 80% of diagnostic procedures and is usually prepared in a ^99^Mo/^99m^Tc generator form using high specific activity (fission moly) or low specific activity (gel moly) Mo-99 loaded on a column shielded in proper lead container to be sent to nuclear medicine centres. The generator systems are then eluted using appropriate aqueous solution and the ^99m^TcO_4_^-^ radio-anion is then mixed with appropriate cold kits containing a ligand and some other additives (reducing agent, stabilizer, filler, …) to obtain an injectable IV solution. Based on the nature of the kit ligands used, the radiopharmaceutical can be used in the diagnosis of various human diseases, such as malignancies, bone problems, heart disorders, liver diseases*, etc.* Table 1 demonstrates a list of available Tc-99m radiopharmaceuticals in the country which have been routinely used in human.

**Table 1:** List of available Tc-99m radiopharmaceuticals in the country

**No**	**Tc–99m tracer**	**Chemical structure**	**Imaging application**
1	Pertechnetate	Small anion/precursor	Thyroid scan; Cystography; Dacryoscintigraphy; Meckel’s diverticulum; Salivary gland imaging; Testicular scan
2	^99m^Tc-MIBI	Small molecule	Myocardial perfusion scan; Parathyroid scan
3	^99m^Tc-MDP	Small molecule	Bone scan
4	^99m^Tc-DMSA	Small molecule	Renal cortical imaging
5	^99m^Tc-DTPA	Small molecule	Kidney scan; Gastric emptying; Cisternography; Testicular scan
6	^99m^Tc-MAA	Small molecule	Lung perfusion scan
7	^99m^Tc-EC	Small molecule	Renal function study
8	^99m^Tc-RBC	Radiolabeled cells	Micro bleeding detection; Hepatic hemangioma
9	^99m^Tc-Phytate	Colloid	Liver/Spleen scan; Lymphoscintigraphy
10	^99m^Tc-BRIDA	Small molecule	Hepatobiliary/Gallbladder scan
11	^99m^Tc-ECD	Small molecule	Brain SPECT
12	^99m^Tc-Octreotide	Peptide	*SSTR+ tumors
13	^99m^Tc-Bombesin	Peptide	**GRPR+ tumors
14	^99m^Tc-UBI	Peptide	Infection imaging
15	^99m^Tc-TRODAT-1	Small molecule	Parkinson's disease; Dopamine transporter imaging agent

* Somatostatin receptor

** Gastrin-releasing peptide receptor

Other important diagnostic tracer in form of ^131^I-NaI oral solution or capsules is routinely used in the detection of thyroid related diseases and metastasis usually in low doses, the radioisotope is produced by the irradiation of natural tellurium target in research reactor. At higher doses this radiopharmaceutical is used for the treatment of hyperthyroid disease and targeted therapy of thyroid malignancies.

Another important I-131 based radiopharmaceutical is the synthetic homologue of neuroamines called I-131 *meta*-iodobenzyl guanidine (^131^I-MIBG) used in the diagnosis of neural crest derived tumors, such as neuroblastoma and pheochromocytoma at low doses. At higher dose, this radiopharmaceutical is used for targeted therapy of the neuroendocrine malignancies.

Tl-201 thallous chloride radiopharmaceutical is used in nuclear medicine based on homology to potassium cation incorporating into biological systems specially Na^+^/K^+^ ATPase pumps. Since there is no appropriate potassium radioisotope from the physical point of view to be used for human use, Tl-201 thallous chloride has been considered an important radiopharmaceutical for imaging of organs overexpressing this pump at the cellular level such as myocardial as well as some malignant cells. The radionuclide is produced in a medium sized cyclotron (30 meV) located in Karaj (NRCAM, AEOI) since 1993 and has been used as the gold standard in the myocardial viability test in human.

Another cyclotron based radiopharmaceutical is Ga-67 citrate, usually used in the detection of infections, inflammations and some malignancies based on homology to ferric cation in the biological systems. In-111 radionuclide is an interesting cyclotron product used hugely in radioimmunoscintigraphy research studies and also in small molecules in form of ^111^In-oxinate for the tracing of radiolabeled cells such as stem cells, leucocytes *etc*. or used as ^111^In-DTPA for imaging of cerebrospinal fluid in specific diseases (cisternography).

An interesting ^81^Rb/^81m^Kr generator for pulmonary studies of lung diseases has been also produced since 1996 as a very safe gaseous radiopharmaceutical that could be even used in infants due to ultra-short half-life (13 seconds) Kr-81m radioactive gas.

The most important and recent diagnostic method called PET has been clinically started in the last 4 years in the country, although the research and development on these tracers initiated almost 2 decades ago. The most important PET radiopharmaceutical is F-18 fluorodeoxy-D-glucose (^18^F-FDG) tracer, a miraculously developed tracer in late 1970’s and early 1980’s due to many physical, biochemical and radiochemical properties. The tracer is specifically integrated into GT1+/HK1+ cells of tumors, brain and also heart and since being approved by FDA in 1991 has been used in the detection and follow-up of more than 30 human malignancies as well as cardiac and brain studies. Sodium fluoride-18 (^18^F-NaF), is another important PET tracer, easily binding to hydroxyapatite part of the skeletal system offering a gold standard of bone imaging in the nuclear medicine. The other important series of PET tracers are developed from the exciting ^68^Ge/^68^Ga radioisotope generator providing an in- house gallium-68 production system, letting every single remote PET center around the country to produce in-house radiopharmaceuticals emphasizing the necessity of hospital radiopharmacies. The recent production and evaluation of Ga-68 generator in the country has been reported and initial human studies on prostate cancer using ^68^Ga-PSMA has been also announced. The other internationally used Ga-68 tracers such as ^68^Ga-citrate, ^68^Ga-DOTATATE, ^68^Ga-DOTATOC has also been developed and are ready to enter the clinical evaluations in 3 existing Iranian PET centers.

In case of therapeutic radiopharmaceuticals as important and recently developing part of radiopharmaceutical science and industry in the country, we already noted the two I-131 agents, i.e. ^131^I-NaI and ^131^I-MIBG. The other older agent, P-32 colloid has been used for intracavital treatment of malignancies. P-32 sodium phosphate has been used in the management of polycythemia vera and is effective for the treatment of chronic myelocytic leukemia and chronic lymphocytic leukemia however due to side effects its application is limited.

Recently, ^153^Sm-EDTMP, ^186^Re-HEDP and ^177^Lu-EDTMP were respectively produced using Sm-153 (half-life 1.7 d), Re-186 (half-life 3.7 d) and Lu-177 (half-life 6.7 d) radioisotopes obtained by Tehran Research Reactor and were used in various clinical studies and successful demonstration of bone pain palliation in patients suffering from metastatic irremediable skeletal pains. Soon after in order to back up the supply chain, in case of reactor production discontinuity, the other important bone pain palliative agent; ^188^Re-HEDP was produced from generator based radioisotope (^188^W/^188^Re generator) and successfully went through clinical evaluations.

Another important series of therapeutic radiopharmaceuticals include radiosynovectomy agents used in the significant alleviation of arthritis pains in patients suffering from joint inflammations due to immunologic/pathologic problems. The application of ^90^Y-citrate colloid in the management of large joint radiosynovectomy was successfully shown in separate clinical studies in the country and the agent is at the moment available. Y-90 is a generator produced therapeutic beta emitter obtained from long-lived (30 y) generator available in the country. Also ^188^Re-sulfur colloid is being used for the radiosynovectomy as demonstrated in other clinical evaluations in the country based on its availability via ^188^W/^188^Re generator.

At the moment more than 50 radiopharmaceuticals are available in Iranian Drug List that most of them are produced and used in the nuclear medicine centers at a regular basis and other available per request (4). The research and development for more sophisticated radiopharmaceuticals based on biomolecules such as peptides, antibodies and their fragments are ongoing and related clinical evaluations should be initiated.

Dr. Amir Reza Jalilian is currently working as a Professor of Radiopharmacy at Radioisotope Products and Radiation Technology Section, IAEA, Vienna, Austria. He could be reached at the following e-mail address: a.jalilian@iaea.org

Dr. Davood Beiki is currently working as a Professor of Radiopharmacy at Research Center for Nuclear Medicine, Shariati Hospital, Tehran University of Medical Sciences, Tehran, Iran. He could be reached at the following e-mail address: beikidav@tums.ac.ir
